# Evaluating accession decisions in customs unions: a dynamic machine learning approach

**DOI:** 10.1007/s10368-024-00632-w

**Published:** 2024-10-03

**Authors:** Dominik Naeher, Philippe De Lombaerde, Takfarinas Saber

**Affiliations:** 1 Department of Development Economics, University of Goettingen, Waldweg 26, 37073 Goettingen, Germany; 2 Neoma Business School, Rouen, France; 3 United Nations University Institute On Comparative Regional Integration Studies (UNU-CRIS), Bruges, Belgium; 4 Vrije Universiteit Brussel, Brussels, Belgium; 5 Lero – the Irish Software Research Centre, School of Computer Science, National University of Ireland, Galway, Dublin Ireland

**Keywords:** Regional integration, Customs union, Accession, Machine learning, Dynamic clustering algorithm, C60, F13, F15, F60

## Abstract

Previous work in the literature on regional economic integration has proposed the use of machine learning algorithms to evaluate the composition of customs unions, specifically, to estimate the degree to which customs unions match “natural markets” arising from trade flow data or appear to be driven by other factors such as political considerations. This paper expands upon the static approaches used in previous studies to develop a dynamic framework that allows to evaluate not only the composition of customs unions at a given point in time, but also changes in the composition over time resulting from accessions of new member states. We then apply the dynamic algorithm to evaluate the evolution of the global landscape of customs unions using data on bilateral trade flows of 200 countries from 1958 to 2018. A key finding is that there is considerable variation across different accession rounds of the European Union as to the extent to which these are aligned with the structure of “natural markets,” with some accession rounds following more strongly a commercial logic than others. Similar results are also found for other customs unions in the world, complementing the insights obtained from static analyses.

## Introduction

Customs unions (CUs) have become a popular avenue for regional integration efforts, covering 120 countries in the world economy today.^1^ This landscape has been gradually shaped over time, dating back at least to the establishment of the German *Zollverein* (1834–1919).^2^ Given the widespread use and at the same time large heterogeneity in the formation history of different CUs, it is natural to ask what the economically optimal size and composition of CUs may be, and to what extent the historical evolution of the CU landscape aligns with those features (or not). These questions continue to be at the center of debates among both policymakers and researchers, often motivated by the fact that CUs are still often perceived as incomplete and CU member states still pursue autonomy regarding the negotiation of extra-regional FTAs (e.g., in the cases of Mercosur, Andean Community, and East African Community), while in other cases, CU membership itself is explicitly questioned (e.g., the fierce debate surrounding Brexit). More generally, there is an economic puzzle regarding the optimal size of economic integration groupings, comprising not only the CU literature, but also the literatures on optimum currency areas (OCA) and “natural markets,” defined as a (regional) market characterized by net trade creation which is welfare increasing (Jacquemin and Sapir 1991; Krugman 1991).

Following Viner’s seminal contribution to the conceptualization of CUs (Viner 1950), the empirical analysis of real-world or planned CUs was gradually sophisticated (Sellekaerts 1973). These analyses usually focused on the trade creation, trade diversion, and terms-of-trade effects of specific (pre-defined) CUs. Only recently, the systematic data-driven evaluation of the composition of CUs and other regional arrangements such as monetary unions was added to the research program (Benassy-Quere and Coupet 2005; Tsangarides and Qureshi 2008; De Lombaerde et al. 2023; Saber et al. 2023). To circumvent issues arising from the complexity and large number of potential country compositions, these studies propose the use of network clustering algorithms adapted from the machine learning literature to evaluate regional arrangements. The main idea underlying these approaches is to start with a set of individual countries (or groups of countries) and then iteratively merge those countries that are most integrated with each other according to some criteria (e.g., the criteria implied by the optimum currency area theory). This provides a data-driven way to evaluate to what degree different actual groupings of countries are in line with the structure of “natural markets” observable from the data.

So far, the existing literature has used static algorithms which are designed to evaluate the composition of these regional arrangements at a given point in time. In this paper, we expand upon these static approaches to develop a dynamic framework which, for the first time, also allows for the evaluation of changes in the composition of CUs over time, such as accessions of new member states.

Moving from a static to a dynamic analysis requires several modifications compared to the algorithms proposed in previous studies. First, the algorithms in previous studies typically start with a set of individual countries that have not yet been grouped in any way (a more detailed description of this feature is provided in Sect. 2). In contrast, our dynamic algorithm allows for the input of a set of starting conditions which capture the landscape of customs unions already in force at any point in time. This means that the first step of our algorithm applied to the data for each year does not start with a set of individual countries but accounts for possible groupings of countries stemming from the previous year. This new feature is especially important for a dynamic analysis over longer periods of time, as it ensures that the results for each year are consistent with the evolution of CUs in the real world. In contrast, using the comparative static approach to evaluate accession decisions would lead to misleading (and less relevant) results as it does not take path dependence into account; that is, it would not take the formation of CUs in previous periods into account, based on trade structures in those periods. At the same time, our dynamic algorithm is designed in a way that subsumes the static one; that is, if the algorithm was applied without historical data on the formation of CUs (treating each country as independent and using only the latest trade data), then the dynamic approach reduces to the static one. This feature both ensures the comparability of our results with previous studies regarding the evaluation of the composition of CUs at a given point in time and introduces the new possibility to provide a meaningful evaluation of accession decisions for new member states.

Another novelty of our proposed approach concerns the distance function (metric) that is used to determine how close a country is to each CU according to the empirical trade intensities. These modifications are necessary because the metrics used in the static algorithms mentioned above are usually chosen so that the obtained results are comparable across different CUs in a given year. However, they are not generally comparable across years since they are sensitive to the total number of countries for which data are available, which changes across years. Because one of our goals is to compare different accessions to the same CU (e.g., the various countries that joined the European Union (EU) over time), and these accessions take place in different years, we develop an alternative metric that is less sensitive to the number of countries in the sample for any given year.

To demonstrate the intuition behind and usefulness of our proposed approach, we apply the dynamic algorithm to evaluate changes in the composition of various CUs corresponding to accessions of new members using data on bilateral trade flows of 200 countries in the period 1958–2018.^3^ The results provide a rich set of insights on the forces underlying the evolution of the CU landscape over time, which complement the insights from the static analyses in previous studies. For example, a key finding is that there is considerable variation across different accession rounds of the EU as to the extent to which these are aligned with the structure of “natural markets” captured by the clustering algorithm, with some accession rounds of the EU CU following more strongly a commercial logic than others. When Greece joined the EU in 1981, there were four other countries bordering the EU with a similarly strong link to the EU CU, namely Albania, Libya, Malta, and Yugoslavia. Only Malta and part of Yugoslavia (Slovenia, Croatia) joined the EU later, despite the fact that many of the other countries continued to show strong economic links to the EU CU. We interpret this finding as suggesting that the ongoing exclusion of these countries from the EU is mostly driven by non-trade related considerations. Similar observations can be made for other accessions to the EU as well as for accessions to other CUs. Overall, this allows us to track over time the degree to which the expansion of each CU appears to have followed a commercial logic (in line with the “natural markets” hypothesis based on trade intensities) as opposed to other (e.g., political) considerations.

To some extent, our study also relates to the rich literature on the determinants of free trade agreements (FTAs) which investigates the factors affecting their creation and composition (Baier and Bergstrand 2004; Egger and Larch 2008; Chen and Joshi 2010; Deltas et al. 2012). The distinct character of CUs compared to FTAs is mainly related to the following.^4^ A CU allows countries to eliminate intra-regional border controls, which is not the case in an FTA. It is therefore that CUs are, in principle, formed among neighboring countries, which is not generally the case for FTAs. An alternative explanation of this intra-regional orientation of CUs, but leading to a similar conclusion, has been given by Lake and Yildiz (2016) who show that rising transportation costs in trade relations with non-neighboring partners (outweighing the benefits of receiving preferences) can be compensated by the benefits of entering additional agreements with third countries simultaneously. This is possible in the case of FTAs but not in the case of CUs. The existence of a common external tariff, in addition, is logically inconsistent with overlapping arrangements, which typically characterize FTAs (but not CUs). Finally, the creation of a CU also implies that countries agree on an institutional mechanism to redistribute tariff revenues collected at the external borders, independently of the level of external protection, which is not needed in the case of FTAs.

This article does therefore not deal with the “choice” countries might be facing between entering an FTA or CU, but assesses the composition of CUs (and its dynamics over time) once countries have decided to form a CU, with all its implications. In line with the above, our proposed algorithm is constructed in a way that makes it suitable for studying non-overlapping groups of countries (such as the CU landscape) but not overlapping arrangements (such as FTAs).

The rest of the paper is organized as follows. Section 2 provides additional background on existing network clustering algorithms and then presents a dynamic machine learning approach to evaluate accession decisions in CUs. Section 3 describes the application and underlying data sources. Section 4 presents the empirical results. Section 5 concludes.

## Methodology

Our analysis focuses on assessing the extent to which CUs are in line with economic considerations according to the prevailing “natural markets” (i.e., member countries of a CU benefit a lot from the CU because they trade heavily with each other) or driven by other factors such as political considerations (i.e., member countries of a CU trade relatively little with each other). For this purpose, we define “natural markets” as markets that maximize the normalized intra-regional market trade shares in an iterative clustering process. This concept has been proposed earlier as an international market characterized by net trade creation which is net welfare increasing (Jacquemin and Sapir 1991; Krugman 1991), although no consensus has been reached so far regarding the choice of the empirical criterion to identify such markets.

Specifically, we expand upon the static clustering algorithms used in the existing literature (Benassy-Quere and Coupet 2005; Tsangarides and Qureshi 2008; De Lombaerde et al. 2021) to develop a dynamic framework which provides data-driven evidence on accession decisions in CUs over time. In the following subsections, we first provide some background on the intuition underlying the use of network clustering algorithms for evaluating CUs and define relevant concepts, and then explain our proposed dynamic methodology.

### Background: Hierarchical clustering with static analysis

Consider the static problem of evaluating the composition of CUs at a single point in time. In the literature quoted above, this is done by applying a hierarchical network clustering algorithm along the following steps. Starting with an initial list of all countries $C=\{{c}_{1}, {c}_{2}, ..., {c}_{n}\}$ and their set of borders$B$, where each country constitutes its own “region,” the algorithm iteratively merges those pairs of bordering regions $r$ that are associated with the largest integration scores $S$ (measured by the normalized intraregional trade shares) among all possible pairs of bordering regions:

1 $$S\left(r\right)=\frac{{\sum }_{\left({c}_{i},{c}_{j}\right)\in {r}^{2},{c}_{i}\ne {c}_{j}}T r a d e\left({c}_{i},{c}_{j}\right)}{\left({\sum }_{{c}_{i}\in r}{\sum }_{{c}_{j}\in C}T r a d e\left({c}_{i},{c}_{j}\right)\right)\bullet \left({\prod }_{{c}_{i}\in r}G D P\left({c}_{i}\right)\right)}$$

where $Trade\left({c}_{i},{c}_{j}\right)$ is the sum of exports and imports between countries ${c}_{i}$ and ${c}_{j}$, and $GDP\left({c}_{i}\right)$ is country ${c}_{i}$’s gross domestic product.^5^ The outcome after $s$ iterative steps (i.e., $s$ times merging two regions) is a set of endogenously determined, non-overlapping groups of bordering countries called regional integration clusters (RICs, or simply “regions” for short).

Definition 1. Regional integration clusters of countries $C$ at step $s$ are a set of regions,$RICs=\{{r}_{1}, {r}_{2}, .., {r}_{k}\}$, such that:

2 $$\forall \left({r}_{k},{r}_{l}\right)\in {RICs}^{2}, {r}_{k}\ne {r}_{l}, {r}_{k}\cap {r}_{l} = \varnothing$$

3 $${\cup\;}_{r\in RICs}r=C$$

Because the RICs are obtained by iteratively merging those pairs of bordering regions that are associated with the largest integration scores $S$, they capture the structure of the regional economic linkages in the data. The RICs can thus be thought of as a hypothetical benchmark grouping representing those groups of countries that are most integrated with each other according to the considered measure of regional trade integration. In a final step, using the RICs as a benchmark offers a way to evaluate how adequate regional arrangements are from an economic perspective; that is, how much real-world CUs are aligned with the empirical trade intensities among the participating countries.

### Assessing the composition of cus over time

Before investigating individual accessions to a CU, we next consider a method for assessing the evolution of the composition of CUs over time. In essence, this approach consists in repeating a cross-sectional analysis for each year in the considered time period. The underlying idea thus is to apply the same static algorithm as described above to each year of an annual dataset of countries’ bilateral trade flows, and measure the distance between each CU and its closest counterpart region (RIC) in the hierarchical clustering for each year. For this purpose, the distance between a CU and its benchmark RIC (i.e., the *Minimum distance to RIC*) is defined as the number of countries that need to be removed from, plus the number of countries that need to be added to the CU to convert it into its closest benchmark RIC. Formally, the *Minimum distance to RIC* for a given region ${r}_{CU}$ can be written as

4 $$d\left({r}_{CU},{r}_{RIC}^{*}\right)=\left|{r}_{CU}\backslash {r}_{RIC}^{*}\right|+\left|{r}_{RIC}^{*}\backslash {r}_{CU}\right|,$$

where $|x|$ is the number of elements (countries) in region$x$, $"\backslash "$ denotes the set subtraction operator (recall that each region is a set of countries), and ${r}_{RIC}^{*}$ is the regional cluster with the closest distance to ${r}_{CU}$ among all$RICs=\{{r}_{1}, {r}_{2}, .., {r}_{k}\}$. For example, if the distance between regions ${r}_{A}\in CU$ and ${r}_{A}^{*}\in RICs$ is smaller than the distance between ${r}_{B}\in CU$ and${r}_{B}^{*}\in RICs$, then we will interpret this as indicating that the composition of ${r}_{A}$ matches the regional trade linkages in the data better than the composition of${r}_{B}$.

The results from this analysis contribute in two ways to the existing literature. First, the analysis of CUs in previous work only considers the composition of CUs at a single point in time (e.g., the year 2018 in De Lombaerde et al. 2021). The analysis presented here extends the results to each year of the period between 1958 (the year when the first CU entered into force) and 2018, which allows us to track the performance of each CU over time. Second, it also allows us to study the composition of each CU at its moment of entry into force (instead of focusing only on the most recent composition in 2018), which is of particular interest for comparative analyses of the historical development of the CU landscape.

### Hierarchical clustering with dynamic analysis

We now turn to the description of our proposed dynamic network clustering algorithm which allows for the evaluation of changes in CU composition (i.e., accessions) over time.

Algorithm 1 provides the pseudocode of the dynamic clustering algorithm. For a given initial list of regions and a list of countries, the algorithm starts by defining a regional configuration that consists of (i) a set of initial regions consisting of multiple countries each, and (ii) other countries (each forming its own region). Then, the algorithm iteratively finds the best two bordering regions in the latest configuration (based on their regional integration score $S\left(r\right)$) before merging them, yielding a new (and more integrated) regional configuration. The algorithm repeats this process until ending up with a final configuration that is only composed of a single region (comprising all countries).

**Algorithm 1**: Dynamic clustering algorithm.
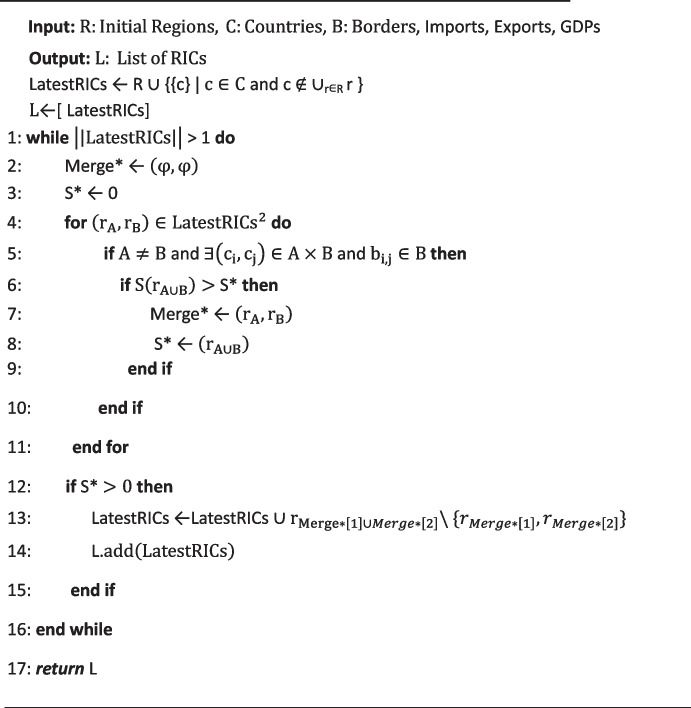


Notice that there are two sets of modifications involved when moving from a static to a dynamic analysis: (i) on the clustering algorithm itself and (ii) on the evaluation criteria (distance functions) used to determine how close countries are to RICs.

Recall that the static clustering algorithm described above starts by considering a list of regions ${r}_{\left\{{c}_{i}\right\}}$, each consisting of a single country ${c}_{i}\in C$ (i.e., each country forms its own region). In contrast, the dynamic algorithm (Algorithm 1) allows for a set of initial conditions, that is, starting with a set of pre-defined regions while retaining the basic logic behind the merging strategy. This new feature makes it possible to not only emulate a fully data-driven regional grouping (i.e., by starting the clustering with independent countries), but also to assess the potential evolution of existing regions over time (by specifying the landscape of CUs in force in any given year as a starting condition). Furthermore, the dynamic clustering algorithm keeps track of all the generated RICs throughout the iterative steps until reaching a unique configuration that composes all the considered countries.

The second set of modifications concerns the underlying evaluation criteria (distance functions). To evaluate and compare accessions to different CUs at different points in time, a distance function (metric) is used which measures how close countries are to each regional cluster according to the observed trade intensities. We consider two different metrics to measure the distance between a CU and an accession country: the *Total distance* and the *CU-specific distance*.

The *Total distance* between a RIC and a candidate country is defined as the step of the clustering algorithm in which the candidate country gets merged with the RIC representing the CU, divided by the total number of steps in the respective year. While the *Total distance* measure is comparable across different CUs in a given year, it remains sensitive to the total number of countries (which varies across years depending on data availability). Therefore, the *Total distance* is not generally comparable across years.

Given that one of our goals is to compare different accession candidates for the same CU (e.g., the various countries that joined the EU over time), and these accessions take place in different years, we also define a second distance measure which is less sensitive to the total number of countries. Specifically, the *CU-specific distance* is defined as the number of times the RIC representing the CU gets merged in the clustering algorithm until it is merged with the candidate country, divided by the total number of times the RIC gets merged in the respective year. Because the *CU-specific distance* only considers those steps of the clustering algorithm involving the respective CU, it is independent of the number of countries in the sample located in geographical parts of the world that are sufficiently far away from the CU of interest, that is, that are not bordering the CU of interest nor any of its accession candidates.^6^

## Data and application

To demonstrate the usefulness of our proposed dynamic clustering algorithm, we now apply the methodology described in the previous section to evaluate the evolution of real-world customs unions. Specifically, we are interested in assessing the extent to which the composition of each CU in each year can be viewed as “natural” (i.e., in line with the observed trade integration intensities, and opposed to being driven by other factors such as political considerations), and how this feature changes over time when new CUs are created and existing CUs take on new members. For this purpose, the first part of the empirical analysis assesses the composition of CUs in each year using the adapted static approach described in Sect. 2.2. The second part of the analysis uses the dynamic algorithm described in Sect. 2.3 to assess the accessions to each CU.

It is important to note that the analysis is of a descriptive nature and is not designed to estimate counterfactual outcomes or make predictions about the outcomes that would have emerged under alternative CU accession scenarios. For instance, the analysis does not provide results that would allow to make statements such as “had country A not joined the EU in 2004, then its trade flows with country B in 2010 would have been larger/smaller than they actually were.” When interpreting the results, it should furthermore be noted that the results for any given year are solely based on the trade intensities observed in that year, and thus do not capture phasing-in and anticipation effects which might play a role in real-world decisions about CU accessions. At the same time, an additional observation is that if we conclude, for example, that a new accession to an existing CU is at some point in time second-best, then this conclusion remains robust in the presence of anticipation effects, because it would mean that the accession is second-best even if we take the (positive) effect of anticipation on trade flows into account.

**Fig. 1:**
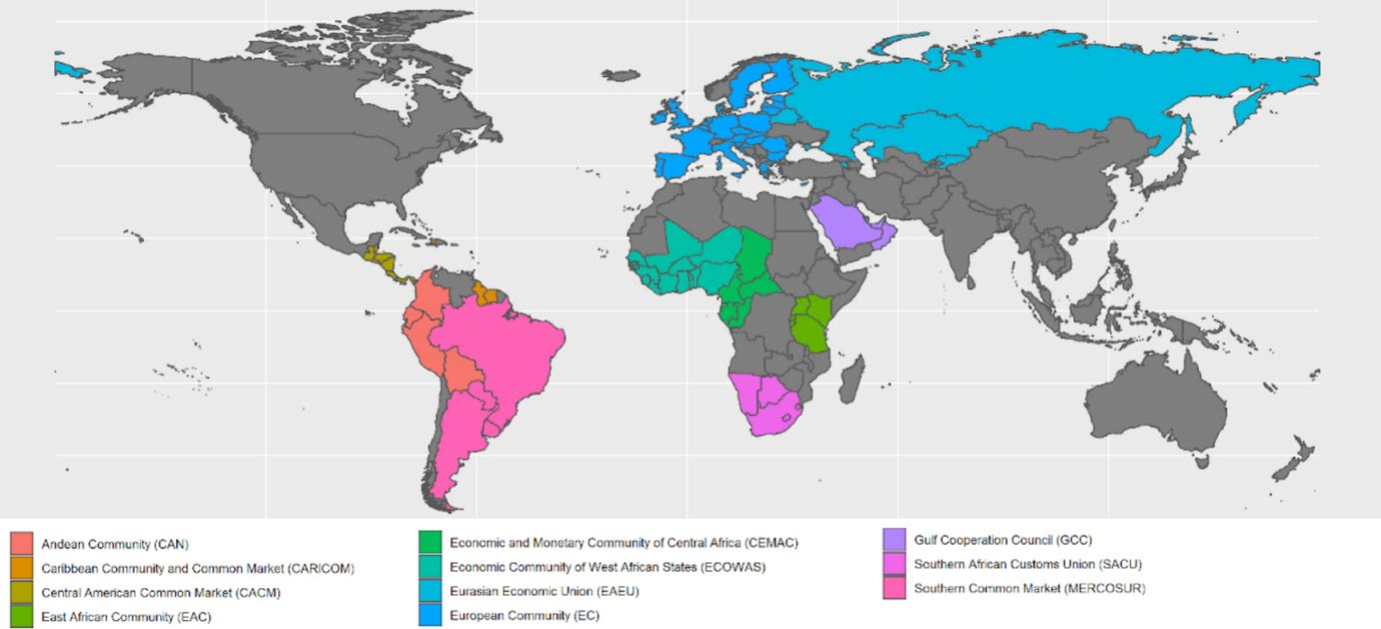
Customs unions as of 2018. Countries in grey were not a member of any of the considered customs unions in the year 2018

Our application focuses on the period from 1958 (the year in which the European Community came into force forming the first CU) to 2018 (the latest year for which data was available when the research project started). We study 11 CUs that were in force during at least some time in this period (see Table 1).^7^ Figure 1 provides a map showing the geographical expansion of these CUs in the year 2018. As it becomes immediately clear from this map, CUs are not homogeneously distributed across world regions. In some geographical areas (such as South America, Europe, and parts of Africa), CUs are much more prevalent than in other areas (Asia, North America). At the same time, it is important to note that these latter areas feature other arrangements aimed at fostering economic integration, such as a large number of FTAs in Asia and North America, which may act as (partial) substitutes for the creation of CUs (see the discussion on the driving forces behind the choice between FTAs and CUs; e.g., Lake and Yildiz 2016; Facchini et al. 2021). Our analysis focuses exclusively on CUs, with a proposed clustering algorithm that is not applicable to overlapping arrangements such as FTAs, for the reasons discussed before.

**Table 1:** Composition of customs unions in year of entry into force

Customs union	Acronym	Countries	Size	Entry into force
Andean Community	CAN	Bolivia, Ecuador, Colombia, Peru	4	1988
Caribbean Community and Common Market	CARICOM	[Antigua and Barbuda], Bahamas, Barbados, Belize, [Dominica], Grenada, Guyana, Haiti, Jamaica, [Montserrat], [Saint Kitts and Nevis], [Saint Lucia], Saint Vincent and the Grenadines, Suriname, Trinidad and Tobago	10	1973
Central American Common Market	CACM	El Salvador, Guatemala, Nicaragua	3	1961
East African Community	EAC	Kenya, Tanzania, Uganda	3	2000
European Community	EC	[Belgium], France, Germany, Italy, [Luxembourg], Netherlands	4	1958
Economic and Monetary Community of Central Africa	CEMAC	Cameroon, Central African Republic, Chad, Congo, Equatorial Guinea, Gabon	6	1999
Economic Community of West African States	ECOWAS	Benin, Burkina Faso, Cabo Verde, Côte d'Ivoire, Ghana, Guinea, Guinea-Bissau, Liberia, Mali, Niger, Nigeria, Senegal, Sierra Leone, The Gambia, Togo	15	1995
Eurasian Economic Union	ECOWAS	Belarus, Kazakhtan, Russian Federation	3	1997
Gulf Cooperation Council	GCC	Bahrain, Kuwait, Oman, Qatar, Saudi Arabia, United Arab Emirates	6	2003
Southern African Customs Union	SACU	Botswana, Eswatini, Lesotho, Namibia, South Africa	5	2004
Southern Common Market	MERCOSUR	Argentina, Brazil, Paraguay, Uruguay	4	1991

All data used in this study are openly available from the following sources. Data on bilateral trade flows come from the IMF’s Direction of Trade Statistics (IMF 2021) which contains annual export and import flows since 1950. For each year and country pair, total trade is calculated as the sum of exports and imports (where two countries report different values for their trade flows, the mean is used). Data on GDP, which is used for normalizing the intraregional trade shares, come from the United Nations Statistics Division (UN 2021) for the period 1970–2019, and from the Penn World Table version 10.0 (Feenstra et al. 2015) for the period 1958–1969. The combination of different data sources is necessary to maximize the number of countries with available data, which is crucial for the robustness of our analysis.^8^ Overall, the sample includes up to 200 countries covering the period from 1958 to 2018.

## Empirical results

This section presents the results of the two parts of our empirical analysis, starting with the assessment of CU compositions over time and then evaluating individual accession decisions for each CU.

### Assessment of CU compositions (static analysis)

**Fig. 2:**
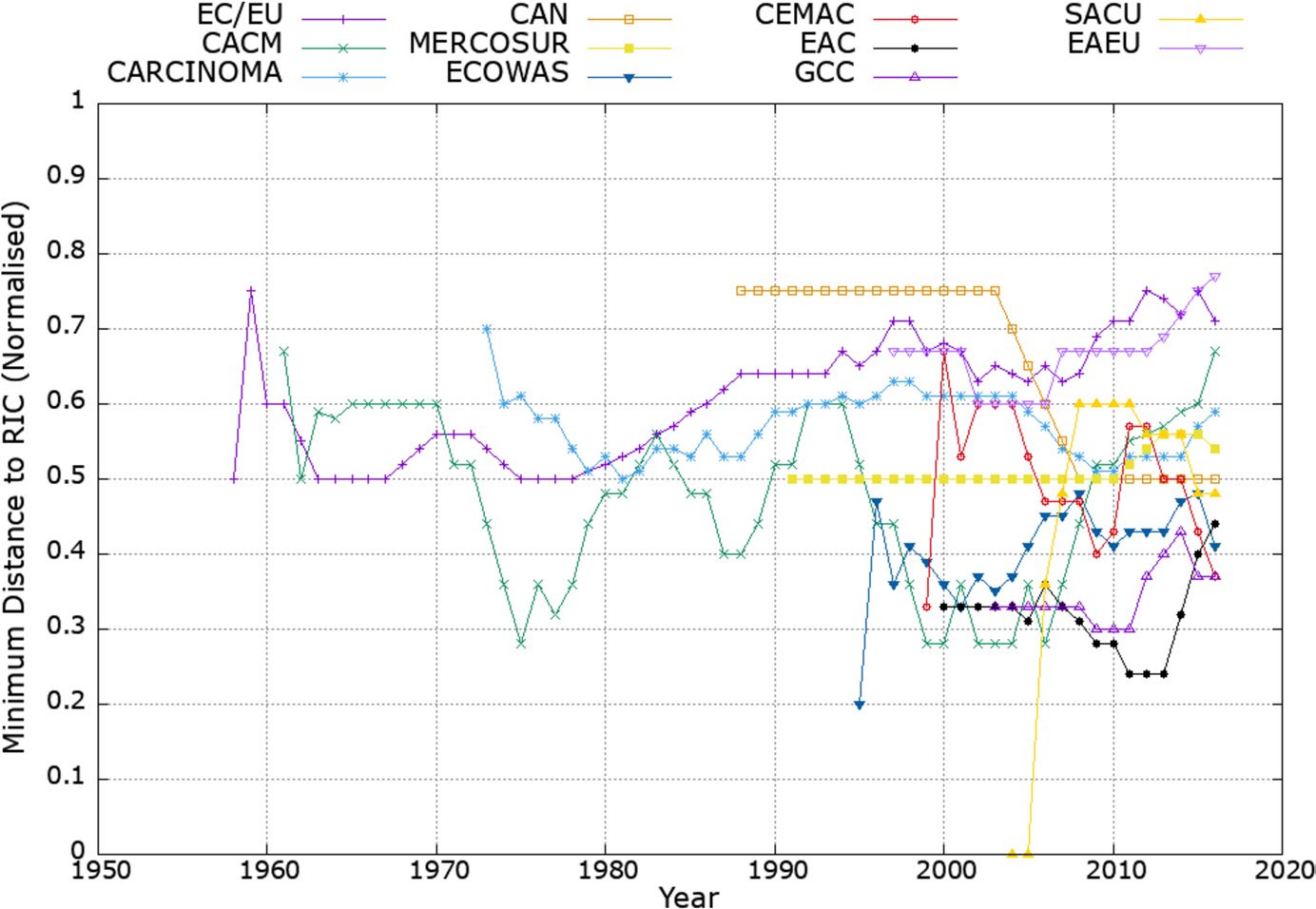
Development of the distance between CUs and RICs over time. The minimum distance to RIC (normalized) is defined as the number of countries that need to be removed plus the number of countries that need to be added from the CU to convert it into the closest RIC, divided by the number of countries in the CU. Depicted values are equally weighted five-year moving averages, including two lags and two leads for each year

Figure 2 shows the evolution of the distance between each CU and the closest RIC (*Minimum distance to RIC*) over time. Recall that a smaller distance indicates a higher match between the composition of the CU and the regional trade linkages in the data which proxy for the structure of “natural markets.” Thus, CUs with a smaller distance score can be interpreted as being relatively more driven by natural economic (trade-related) forces as opposed to other factors (including political considerations). The distance of each CU fluctuates over time both because of changes in the CU composition (e.g., accessions) and because of changes in the underlying trade flows between countries.

According to the results in Fig. 2, there is considerable variation across CUs as to their distance to the RICs emerging from the clustering algorithm, suggesting that some CUs are relatively more driven by “natural” economic forces than others. For example, over the past 20 years, the European Union (EU) and the Eurasian Economic Union (EAEU) featured distance scores above 0.6, whereas the East African Community (EAC) and the Gulf Cooperation Council (GCC) featured distance scores well below 0.4 in most of the years. Combined with the positive trend in the distance score of the EU between the 1970s and 2018, this suggests that at least some of the latest accessions to the EU were driven by non-trade-related (e.g., political) factors rather than by purely trade-related considerations. Of course, the analysis underlying Fig. 2 provides no insights regarding which accession countries this applies to (especially because in the accession rounds of 2004 and 2007 multiple countries joined the EU in the same year). This question will be addressed in detail by the dynamic analysis of CU accessions presented in the next section.

Table 2 reports results on the composition of each CU in its year of entry into force. Column (2) shows the minimum distance between the CU and the respective benchmark RIC. Column (3) shows the normalized distance which is obtained by dividing the distance in column (2) by the number of countries in the CU (indicated in Table 1). Column (4) reports the step of the algorithm at which the minimum distance is (first) reached. Column (5) shows, on the one hand, the CU members that are part of the RIC with minimum distance, and, on the other hand, the (posterior) steps at which the other CU members become part of the same RIC. In addition, the RIC with the minimum distance (at the step when the minimum distance is first reached) may contain countries that are not members of the corresponding CU. These countries are reported in column (6).

**Table 2:** Results on the composition of customs unions in year of entry into force

Customs union	Min. distance to RIC	Distance (Norm.)	Step with Min. Dist	CU countries in RIC with minimum distance (step)	Other countries in RIC (step)
(1)	(2)	(3)	(4)	(5)	(6)
CAN	3	0.75	0	Bolivia (35), Peru (35), Ecuador (127), Colombia (158)	Paraguay (84)
CARICOM	7	0.70	67	Grenada (6), Trinidad and Tobago (6), Saint Vincent and the Grenadines (67), Barbados (130), Guyana (130), Suriname (130), Jamaica (147), Belize (147), Haiti (153), Bahamas (153)	Dominican Republic (110), Venezuela (110)
CACM	2	0.67	0	El Salvador (57), Guatemala (57), Nicaragua (86)	Honduras (3), Mexico (33)
EAC	1	0.33	21	Kenya (21), Uganda (21), Tanzania (180)	Burundi (110), Rwanda (110)
EC	2	0.50	23	Germany (23), Netherlands (23), Italy (54), France (54)	UK (41)
CEMAC	2	0.33	111	Cameroon (111), Central African Republic (12), Chad (12), Congo (187), Equatorial Guinea (111), Gabon (187)	Sudan (152)
ECOWAS	3	0.20	163	Mali (16), Senegal (16), Cabo Verde (82), The Gambia (106), Guinea (145), Guinea-Bissau (145), Sierra Leone (145), Togo (163), Benin (163), Côte d'Ivoire (163), Ghana (163), Liberia (163), Burkina Faso (177), Niger (177), Nigeria (177)	-
EAEU	2	0.67	0	Kazakhstan (189), Russian Federation (189), Belarus (197)	-
GCC	2	0.33	132	Bahrain (49), Qatar (49), Saudi Arabia (132), United Arab Emirates (132), Oman (161), Kuwait (191)	Yemen (161), Iran (161)
SACU	0	0	137	Eswatini (41), South Africa (41), Lesotho (94), Namibia (137), Botswana (137)	-
MERCOSUR	2	0.5	42	Argentina (42), Uruguay (42), Brazil (131), Paraguay (144)	Chile (86)

The *Minimum Distance to RIC* is defined as the number of countries that need to be removed plus the number of countries that need to be added from the CU to convert it into the closest RIC. *Distance (Normalized)* is calculated by dividing the values in column (3) by the number of countries in the CU (column 2).

To illustrate how the results in Table 2 can be interpreted, consider the case of EAC as an example. When EAC entered into force in the year 2000, it had three founding members (see Table 1). The minimum distance to its benchmark RIC emerging from the algorithm is reached in step 21 (column 4 in Table 2). In this step, two of the CU members (Kenya and Uganda; see column 5) are part of the RIC, whereas Tanzania is only added later (in step 180). Also, there are no other countries in the benchmark RIC at step 21 (see column 6). Therefore, the distance in column (2) equals 1 (i.e., one country must be added to form EAC), and the normalized distance in column (3) equals 0.33 (or 1/3). At the same time, there are two other countries, Burundi and Rwanda, that become part of the benchmark RIC in step 110 (see column 6), before Tanzania is added in step 180. This suggests that, according to the underlying logic of our algorithm, the CU would have generated greater trade-integration benefits if Burundi and Rwanda had been included in 2000 rather than Tanzania. Interestingly, these two countries indeed joined EAC slightly later, in the year 2007, which might be seen as support for the empirical plausibility of our methodology.

### Assessment of CU accessions (dynamic analysis)

We now turn to the second part of our application which uses the dynamic clustering algorithm described in Sect. 2 to assess CU accessions. In principle, the obtained results allow for an analysis at two levels: (i) inter-CU (i.e., comparing average distances of accessions between different CUs), and (ii) intra-CU (comparing individual accession countries within each CU). Because the number of accessions is very heterogeneous across CUs (ranging from zero accessions for the GCC to 22 accessions for the EC/EU), we focus on interpreting the results of an analysis at the intra-CU level. The natural choice for this type of analysis is to focus on the EC/EU since it is the oldest, largest, and arguably most influential arrangement in the current CU landscape.

Table 3 reports results for each country that became a member of the EC/EU since its creation in 1958 by Belgium, France, West Germany, Italy, Luxembourg, and The Netherlands. The first two columns provide information on the first three neighboring countries of the CU that get merged with the CU in the algorithm (if several countries get merged in the same step, all are reported). Columns (3) to (6) provide information on the accession countries in the respective year. For example, in 1973, three countries joined the EC, namely Denmark, Ireland, and the UK. Column (3) reports the step in which each accession country gets added to the CU in the algorithm. Column (4) shows the associated *Total distance* while column (5) shows the *CU-specific distance*. Finally, column (6) lists the first three neighboring countries of the accession candidate that get merged with the candidate in the algorithm.

**Table 3:** Results on EC/EU Accessions

CU’s perspective		Candidate’s perspective when joining CU			Candidate’s alternative
Accession year	First 3 neighbors that get merged with CU (Step)	Accession country (step merged with CU)	Total distance	CU-specific distance	First 3 neighbors that get merged with candidate (step)
(1)	(2)	(3)	(4)	(5)	(6)
1973	Spain (119) Algeria (119) Malta (119) Tunisia (119)	Denmark (137)	0.99	1	Norway (45) Sweden (94) UK (106)
		Ireland* (137)	0.99	1	UK (48)
		UK (137)	0.99	1	Ireland (48) Denmark (106) Norway (106)
1981	Libya (128) Malta (128) Albania (135) Yugoslavia (135)	Greece (128)	0.92	0.25	Libya (68) Cyprus (103) Italy (128)
1986	Turkey (127) Cyprus (136) Egypt (136) Libya (136) Malta (136)	Portugal (138)	0.99	0.8	Morocco (47) Spain (75)
		Spain (138)	0.99	0.8	Morocco (75) Portugal (75) Algeria (115)
1995	Albania (128) Bulgaria (128) North Macedonia (128) Turkey (128) Croatia (128) Slovenia (128)	Austria (133)	0.96	0.5	Switzerland (52) Czechia (89) Slovakia (89)
		Finland* (133)	0.96	0.5	Sweden (49) Norway (71) Estonia (124) Russia (124)
		Sweden (133)	0.96	0.5	Finland (49) Norway (71) Lithuania (104) Poland (104)
2004	San Marino (107) Switzerland (109) Slovakia (116) Slovenia (116) Albania (116) Bulgaria (116) North Macedonia (116) Croatia (116)	Cyprus (123)	1	1	Lebanon (32) Syria (68) Israel (90)
		Czechia (116)	0.94	0.5	Slovakia (26) Austria (116) Germany (116)
		Estonia (122)	0.99	0.83	Latvia (15) Russia (99) Finland (122) Sweden (122)
		Hungary (116)	0.94	0.5	Slovenia (36) Croatia (70) Romania (105) Slovakia (105) Ukraine (105)
		Latvia (122)	0.99	0.83	Estonia (15) Lithuania (69) Belarus (99) Russia (99)
		Lithuania (122)	0.99	0.83	Poland (39) Latvia (69) Belarus (99) Russia (99)
		Malta (120)	0.98	0.67	Libya (55) Tunisia (55) Italy (120)
		Poland (122)	0.99	0.83	Lithuania (39) Belarus (99) Russia (99)
		Slovakia (116)	0.94	0.5	Czechia (26) Ukraine (84) Hungary (105)
		Slovenia (116)	0.94	0.5	Hungary (36) Croatia (70) Austria (116) Italy (116)
2007	San Marino (107) Switzerland (108) Egypt (112) Turkey (112) Albania (112) North Macedonia (112) Croatia (112) Serbia (112) Ukraine (112) Montenegro (112)	Bulgaria (112)	0.97	0.6	North Macedonia (17) Turkey (87) Romania (97) Serbia (97)
		Romania (112)	0.97	0.6	Serbia (50) Bulgaria (97) Moldova (97) Ukraine (97)
2013	San Marino (104) Switzerland (105) North Macedonia (107) Serbia (107) Albania (107) Montenegro (107)	Croatia (107)	0.94	0.5	Bosnia & Herzegovina (46) Montenegro (46) Serbia (81)

Prior to the first accession round in 1973, the EC comprised Belgium, France, West Germany, Italy, Luxembourg, and The Netherlands. *Total distance* is calculated as the step of the clustering algorithm in which the candidate country gets merged with the CU, divided by the total number of steps in the respective year. *CU-specific distance* is calculated as the number of times the CU gets merged in the clustering algorithm until it is merged with the candidate country, divided by the total number of times the CU gets merged in the algorithm

*Countries that were not neighboring any member countries of the CU prior to the year of accession

To illustrate how the results in Table 3 can be interpreted, consider the first accession round of the EC in 1973 as an example. At that time, the EC consisted only of its six founding members. The three accession candidates, Denmark, Ireland, and the UK, all get merged with the EC in step 137 of the algorithm. This implies that these three countries had already been part of the same RIC before this step, indicating that their economies were trading relatively strongly with each other at that time. In particular, as can be seen in column (6), Ireland and the UK become part of the same RIC in step 48 of the algorithm, and in step 106, Denmark joins them (together with Norway and possible some other countries which are neither bordering Ireland nor the UK). In addition, column (2) shows that there are four countries (Spain, Algeria, Malta, and Tunisia) that get merged with the EC in an earlier step (119) of the algorithm than the three accession countries of 1973.

These results give rise to several interesting insights. First, they suggest that, from a purely economic perspective, the EC in 1973 would have benefited more from incorporating Spain, Algeria, Malta, and Tunisia compared to taking up Denmark, Ireland, and the UK.^9^ The accession of these three countries can thus be perceived as driven mainly by other factors, including political considerations. Similarly, from the economic perspective of the accession countries, joining the EC was not necessarily the first-best option for them. Instead, the results suggest that Denmark, Ireland, and the UK would have gained more economically by forming a CU together with Norway and Sweden. Interestingly, this is also in line with the composition of the European Free Trade Association (EFTA) at that time, which constituted a free trade area (albeit not a full CU) including the UK, Denmark, Ireland as well as Norway and Sweden (plus a few others).

Similarly, our method generates a rich set of suggestive results for the other accession rounds of the EC/EU covered in Table 3 as well as for the accessions to other CUs reported in Table 4. While it would go beyond the scope of this paper to discuss all of these results in detail, the following list provides some interesting findings that emerge from the analysis.

- When Greece joined the EU in 1981, there were four other countries bordering the EU with a similarly strong link to the EU CU, including Albania and Yugoslavia (as well as Libya and Malta). While part of Yugoslavia (Slovenia, Croatia) joined the EU later, Albania and the rest of Yugoslavia (Bosnia and Herzegovina, (North) Macedonia, Montenegro, Serbia) did not. The fact that these countries continue to show strong links to the EU CU throughout 2007 and 2013 (the last two EU accession rounds) suggests that their ongoing exclusion from the EU may be mostly driven by non-economic considerations.
- San Marino and Switzerland are repeatedly among the first countries that get merged with the EC/EU in the algorithm in recent years, suggesting that the reluctance of Switzerland to join the EU must be mainly driven by other, non-trade related considerations. Additionally, it is also possible that the EU-Switzerland FTA of 1972 acts as a partial substitute for joining the EU CU. The adhesion of San Marino to the EC/EU CU was signed in 1991 and entered into force in 2002.
- In the 1970s and 1980s, the non-EC/EU countries that were most closely linked to the EC/EU economically included many North African countries (such as Algeria, Tunisia, and Libya). In more recent years, countries in Eastern Europe seem to have taken this place (including Albania, Montenegro, North Macedonia, and Serbia), which is interesting since these are also among the recognized candidates for membership of the EU (in addition to Turkey).
- At their time of accession to the EU, the island countries Cyprus and Malta were economically most strongly linked to other non-EU countries in their respective region (i.e., Lebanon, Syria, and Israel in the case of Cyprus, and Libya and Tunisia in the case of Malta), which points to their potential vulnerability in light of the recent conflicts in some of these countries.
- When Honduras joined the CACM in 1962, three other countries (Costa Rica, Panama, and Colombia) were relatively more strongly integrated with the CACM members. This suggests that the subsequent accessions of Costa Rica (in 1963) and Panama (in 2013) strengthened the alignment of this CU with the structure of “natural markets” in Latin America. According to this logic, the next candidates for joining CACM would include Belize, Colombia, Cuba, Jamaica, and Mexico.
- When Venezuela joined MERCOSUR in 2013, there were four other countries (Chile, Bolivia, Colombia, and Peru) that were at least as strongly integrated with the MERCOSUR CU as Venezuela (all these now enjoy the status of an associate country of MERCOSUR). At the same time, Venezuela was relatively more strongly linked to other countries (including Colombia and the Dominican Republic). This suggests that the economic cost associated with the suspension of Venezuela in 2006 may have been relatively limited for both sides.

**Table 4:** Results on Other CU accessions

CU’s perspective		Candidate’s perspective when joining CU			Candidate’s alternative
CU, accession year Members	First 3 Neighbors that get merged with CU (step)	Accession country (step merged with CU)	Total distance	CU-specific distance	First 3 neighbors that get merged with candidate (step)
(1)	(2)	(3)	(4)	(5)	(6)
**CACM, 1962** El Salvador Guatemala Nicaragua	Costa Rica (59) Panama (59) Colombia (77)	Honduras (82)	0.93	0.75	Jamaica (40) Mexico (68) El Salvador (82) Guatemala (82) Nicaragua (82)
**CACM, 1963** El Salvador Guatemala Nicaragua Honduras	Panama (65) Mexico (75) Colombia (85) Jamaica (85)	Costa Rica (65)	0.74	0.33	Panama (6) Nicaragua (65) Colombia (85) Ecuador (85)
**CACM, 2013** El Salvador Guatemala Nicaragua Honduras Costa Rica	Mexico (92) Cuba (92) Belize (101) Jamaica (101)	Panama (108)	0.95	0.6	Colombia (73) Costa Rica (108) Nicaragua (108)
**EAC, 2007** Kenya Tanzania Uganda	Comoros (84) Seychelles (84) Ethiopia (98) Somalia (98) Sudan (98)	Rwanda (114)	0.98	0.83	Burundi (6) Congo, DRC (61) Tanzania (114) Uganda (114)
		Burundi (114)	0.98	0.83	Rwanda (6) Congo, DRC (61) Tanzania (114)
**EAEU, 2015** Belarus Kazakhstan Russia	Norway (67) China (84) Mongolia (84) Japan (106)	Armenia (111)	1	1	Georgia (12) Azerbaijan (62) Iran (62) Turkey (82)
		Kyrgyzstan (111)	1	1	Tajikistan (16) Uzbekistan (44) China (111) Kazakhstan (111)
**MERCOSUR, 2013** Argentina Brazil Paraguay Uruguay	Chile (74) Bolivia (97) Colombia (97) Peru (97)	Venezuela (97)	0.85	0.4	Dominican Republic (59) Aruba (59) Colombia (88) Brazil (97)

*Total distance* is calculated as the step of the clustering algorithm in which the candidate country gets merged with the CU, divided by the total number of steps in the respective year. *CU-specific distance* is calculated as the number of times the CU gets merged in the clustering algorithm until it is merged with the candidate country, divided by the total number of times the CU gets merged in the algorithm

We will not develop these cases further here, nor the results for other CU accessions reported in Table 4. Rather, we simply point out the demonstrated capacity of our methodology to lead to the formulation of interesting hypotheses for further study.

## Robustness and limitations

One concern with the empirical findings presented above may be their robustness to alternative modelling choices in constructing the dynamic clustering algorithm. Since the algorithm is designed as a natural extension of the static approach used in previous studies, its construction largely mirrors modelling choices already established in the literature. Most of these choices are in fact basic operations for hierarchical clustering algorithms which are standard in the machine-learning literature. In addition, our proposed algorithm has the two key features that (1) only countries are merged that share a common border, and (2) the algorithm focuses on maximizing the integration scores of each region as measured by the normalized intraregional trade shares in our empirical application. As highlighted above, intraregional trade shares are a commonly used measure of regional integration in the literature (Capannelli et al. 2009; Naeher and Narayanan 2020; Huh and Park 2021). Nevertheless, it should be noted that this approach focuses exclusively on trade integration and does not allow to capture all the economic benefits typically associated with CUs. We thus cannot rule out the possibility that using different indicators to measure regional integration would lead to different conclusions. Our findings should therefore be seen as providing one among several possible benchmarks for evaluating the composition of CUs from an economic perspective.

Another limitation of our proposed algorithm is that it can currently only be applied to non-overlapping groups of countries. While this limitation is self-imposed for the purpose of studying the CU landscape, it makes it unsuitable for studying other common arrangements aimed at fostering economic integration, such as FTAs, which are often highly overlapping. It would be technically possible to relax this limitation to allow for overlapping arrangements (Whang et al. 2018) and apply the algorithm to a broader set of trade agreements. However, as with most overlapping hierarchical algorithms, this adaptation would require either the introduction of a new fuzzy objective (i.e., fuzzy regional integration) or the addition of some assumptions/constraints regarding the merging threshold, the merging levels, or the geometry of the expected merging graph (N’Cir et al. 2015). Furthermore, it would also require a different conceptual framing and a different set of research questions.

## Conclusion

This study is in many ways a natural continuation of the analyses presented in previous studies that use network clustering algorithms to evaluate regional economic arrangements. Specifically, we expand the static evaluation of the composition of such arrangements at a single point in time to a dynamic framework that allows for the evaluation of changes in composition over time.

We apply the new dynamic method to evaluate CU accessions in a sample of 200 countries over the period 1958–2018. Our results show that there is considerable variation across CUs in a given year, and across rounds of accessions for the same CU, as to the distance to the RICs emerging from the clustering algorithm. This suggests that the expansion of the EC/EU CU, as well as of other CUs, does not always follow a strictly commercial logic aligned with the structure of “natural markets” captured by the clustering algorithm. Our proposed method also generates a rich set of results that can form the basis of case studies of individual CUs, although a more detailed analysis along this line is left for future research.

Another interesting avenue for future research may consist in generalizing the dynamic clustering algorithm so that it becomes applicable to overlapping groups of countries. This would allow for empirical studies of common arrangements aimed at fostering economic integration other than CUs, notably FTAs. As discussed above, this would require the development of several new technical features as well as an adapted conceptual framing. Clearly, more research to assess the optimal composition of trade agreements and guide policy decisions is warranted. Our modelling insights may provide a useful point of departure for such efforts.

## Data Availability

All data used in this study are openly available from the cited sources.

## Footnotes

1 At the start of 2022, WTO data show 29 CUs in force. When accessions and enlargements are filtered out, 13 CUs are counted, covering 120 countries globally. See https://rtais.wto.org (last accessed: April 2023).

2 Early (pre-WWII) CUs also included the ones between France and Monaco (1865-), Belgium and Luxembourg (1921-), and Switzerland and Liechtenstein (1924-). In Latin America, as early as 1909 plans were proposed to create a South American CU, followed by proposals to create Pan-American and Argentine-Brazilian CUs (Porcile 1995; Cramer 1998; Briceño Ruíz and De Lombaerde 2018).

3 This was the maximum period for which data was available at the time we conducted the study. Extending the study period beyond 2018 would not add insights as the most recent accessions to CUs took place in 2015 (accessions of Armenia and Kyrgyz Republic to the Eurasian Economic Union). Changes in the opposite direction (i.e., members leaving CUs) are rare in practice. In our sample period 1958–2018, there is only a single country (Venezuela) to which this applies. The results of our analysis focusing on CU accessions are not affected by this. Since January 2021, the UK is no longer part of the EU CU. The new EU-UK Trade and Cooperation Agreement was provisionally applied as of January 1, 2021, and entered into force on May 1, 2021.

4 Although we do not suggest that combining FTAs and CUs in one analytical model would not make sense for certain purposes, a distinct focus on CUs, as in this paper, without incorporating FTAs simultaneously in the analysis, is justified and needed because CUs and FTAs are not just alternative modalities to reach a given goal (trade liberalization). In a purely (normative) theoretical setting, strict CU formation has also been analyzed separately (Yi 1996).

5 Intraregional trade shares are commonly used in the literature to measure regional integration (Capannelli et al. 2009, Naeher 2015, Naeher and Narayanan 2020, Huh and Park 2021).

6 Note that the results may still be affected by missing data for countries that are bordering the CU of interest or its accession candidates.

7 The analysis includes CUs that are notified to the WTO and currently (as of October 2020) in force. Note that we do not include the EU-Andorra CU and EU-San Marino CU. COMESA is not included because, although its CU was formally notified to the WTO in 1995 under the enabling clause, it is still not operational. The Russian Federation-Belarus-Kazakhstan CU (entry into force: 03/12/1997) is considered as the precursor of the EAEU. The West African Economic and Monetary Union (WAEMU) is not included as all its members are also part of ECOWAS. The EU-Turkey CU is not included as it is only a partial agreement, covering only industrial goods, and without a coherent common external tariff (De Lombaerde and Ulyanov 2020).

8 Where countries split or were unified over the considered period, values were adjusted (as far as possible) to be consistent across different datasets and years. This affected Ethiopia, Eritrea, Sudan, and South Sudan. In all other cases (e.g., the countries forming Yugoslavia and the Soviet Union), the used data sources already provided comparable information.

9 Note that this statement is based solely on the trade intensities observed in 1973, not on any predictions or estimated counterfactual outcomes in later years. In particular, the analysis does not capture phasing-in and anticipation effects which might play a role in real-world decisions about CU accessions (see the discussion in Sect. 3).
